# Recent Progress in Sleep Quality Monitoring and Non-drug Sleep Improvement

**DOI:** 10.3389/fnhum.2020.00021

**Published:** 2020-04-07

**Authors:** Jing Chi, Wei Cao, Yan Gu

**Affiliations:** ^1^Shenzhen Qianhai Icecold IT Co., Ltd., Shenzhen, China; ^2^Center for Stem Cells and Regenerative Medicine, Key Laboratory of Tissue Engineering and Regenerative Medicine of Zhejiang Province, Zhejiang University School of Medicine, Hangzhou, China

**Keywords:** insomnia, sleep disorders, sleep quality, sleep monitoring, polysomnography, light pollution

## Abstract

Insomnia is one of the most common health risk factors in the population as well as in clinical practice, which is associated with genes, neuron, environment, behavior, and physiology, etc. This review summarizes the recent progress in sleep quality monitoring and non-drug sleep improvement. The innovation of wearable and effective invention suggests a new approach and have deep implications toward sleep improvement and yet, the health care innovation system is also facing the challenge to foster the progress.

## Introduction

Insomnia is one of the most common health risk factors in the population as well as in clinical practice, which is associated with genes, neurons, environment, behavior, physiology, etc. (Buysse, 2013; Harvey et al., 2014). Insomnia is defined as the subjective perception of difficulty with sleep initiation (over 30 min of sleep latency), duration, and consolidation, resulting in dissatisfied sleep quality despite adequate opportunity for sleep (Schutte-Rodin et al., 2008). The understanding of the whole dynamics of the sleep–wake cycle could lead us to a better solution on insomnia, which is controlled by interactive neurochemical processes among multiple neural structures (Espana and Scammell, 2004; Brown et al., 2012).

Insomnia has gradually become a prevalent phenomenon in fast-paced urban life. Insomnia occurs among individuals of different ages (Johnson et al., 2006; Kryger, 2006), and symptoms occur in approximately 33–50% of the adult population (Ancoli-Israel and Roth, 1999). Insomnia is the most prevalent and accounts for almost half of all sleep disorders (15% of the whole population) (Cao et al., 2017). Due to the complexity of the neural system that controls sleep, it is a great challenge to accurately diagnose and treat insomnia.

During the past few years, a wide range of hardware including wearable devices has enabled us to access more personal health performance via mobile applications and help improved our health. However, the reliability and validation vary among different applications (Peake et al., 2018). For insomnia management and improvement, it is critical to develop a set of comprehensive sleep valuation as well as following therapies (Kapur et al., 2017).

This review summarizes the recent progress in sleep quality monitoring and non-drug sleep improvement, with a comprehensive analysis on the related advantages and limitations, trying to conclude effective suggestions for sleep problems improvement in general.

## Sleep Quality Monitoring

Insomnia should be properly diagnosed before treatment. Subjective sleep quality assessment is mainly through subtly developed questionnaires. The most commonly used forms are the *Morning Evening Questionnaire (MEQ)*, *Pittsburgh Sleep Quality Index (PSQI)*, *the Hamilton scale*, etc. (Schwab et al., 1967; Horne and Ostberg, 1976; Buysse et al., 1989). PSQI sleep quality assessment was invented by the University of Pittsburgh and was most frequently used. It contains nine questions with each answer scoring between 0 and 3. The PSQI index is calculated as the sum of all the scores. The lower the score is, the better the sleep quality. Clinical studies have shown that the PSQI demonstrates high reliability and validity to analyze sleep problems under many circumstances, but just like other self-report inventories, its scores can be easily affected by the testee (Grandner et al., 2006; Mollayeva et al., 2016).

Sleep doctors routinely use a device called polysomnography recorder (PSG) for sleep quality monitoring (Jafari and Mohsenin, 2010). The PSG records electroencephalogram (EEG), electromyogram (EMG), electrocardiogram (ECG), respiration, and body movements along with other vital signs. Polysomnography is commonly used for sleep quality assessment, therapy, and sleep disorders (Gregorio et al., 2011). There are several limitations to obtain high quality of PSG data, including the first night effect in decreased sleep efficiency due to the unfamiliar environment and lack of comfort brought by the test, the difference in PSG variables by sex and different age groups, the control subjects, research angles and environments of different lab groups, and so on, thus making PSG hard for normalization (Newell et al., 2012; Boulos et al., 2019).

Presently, new technologies and innovations on *wearables* have made sleep monitoring easy to use and enable us to access sleep data in a real-world environment, compared to PSG (Kelly et al., 2012). Recent systematic reviews on the sleep monitoring methods have been introduced to value sleep quality, emphasizing the powerful innovation of wearables and the application on athletes, which allows complementary access with respect to classical sleep quality valuation and diagnose insomnia and its severity level (Peake et al., 2018; Claudino et al., 2019). Comparison on the result between PSG and wearables has shown consistency but needs further refinement for reliability (Lee et al., 2019).

The new Apple watch not only has a sleep monitoring function but also achieved CFDA approval for early warning of atrial fibrillation. A small electrocardiographic device developed by Harvard University infers sleep quality by cardiopulmonary coupling (CPC) monitoring (Thomas et al., 2005). These products are relatively simple in structure when compared to the PSG and are much more comfortable to wear. Most sleep monitoring devices in the consumer market refer to actigraph (body movement), heart rate, and heart rate variability (HRV) to predict sleep structure, to evaluate the quality of sleep (Kosmadopoulos et al., 2014). Other products with comparable functions include wrist bands, sleep monitoring belts, and radar beam trackers. One of such examples is the sleep monitoring belt based on piezoelectric sensing technology developed by an Israeli company named EarlySense and a Finlandizei company called Beddit recently acquired by Apple.

The accuracy of these consumable products, however, has been challenged by medical doctors, and whether they could replace the PSG device for clinical use is yet to be determined. However, a recent trend on the cooperation between the pharmaceutical and wearable companies has been shaping. For instance, Eli Lily and Apple have started large-scale clinical trials on Alzheimer’s disease, with the help of iPhones, watches, and sleep monitor belt.

## Non-Drug Therapy and Sleep Improvement Techniques for Insomnia

Currently, most insomnia patients take hypnotic drugs for treatment. However, a large percentage of the population feel reluctant to take sleeping pills, and this led to the development of non-drug insomnia therapeutics. Non-drug treatment of insomnia is divided into two major categories: cognitive behavioral therapy for insomnia (CBT-I), which is performed in the absence of auxiliary devices (Morin, 2004), and physiotherapy through repetitive transcranial magnetic resonance (rTMS), white noise and music, aromatherapy, and light therapy devices.

*Cognitive behavioral therapy (CBT)* is a fairly simple and easy way to treat insomnia. It can relieve insomnia through such behavioral interventions as sleep restriction, stimulus control, and paradoxical intervention. For example, sleep restriction allows a subject to stay in bed only when he or she feels sleepy and this gradually improves sleep quality by increasing sleep efficiency (Miller et al., 2014). Stimulus control improves sleep quality by limiting non-sleep behaviors in the bed and establishing good bed-sleep conditioned reflexes (Hood et al., 2014). No auxiliary devices are needed for CBT-I, but the drawback of this tool is its low compliance rate.

*Repetitive transcranial magnetic stimulation (rTMS)* relieves insomnia by lowering the level of arousal for the targeted cerebral cortex (Jiang et al., 2013). Low-frequency (<1 Hz) repetitive transcranial stimulation inhibits the excitement of the cerebral cortex and can induce slow waves, a brain wave that mostly appears as a subject enters deep sleep. Although this method is effective, the equipment is too large for consumable commercialization.

*White noise* refers to the combination of sound of multiple frequencies. White noise diminishes the excessive concentration of attention to relax the mood and alleviate insomnia (Messineo et al., 2017). Most natural sounds such as wind, rain, water flow, and other natural sounds all belong to the white noise family. White noise is often used in conjunction with soothing music to regulate mood and help with sleep.

*Aromatherapy* and *meditation* are commonly used together. Both methods are considered to be effective in reducing psychological stress. The fragrance of natural flowers and plants has been accompanying human beings into dreams throughout the 2 million years of evolution. We used to live in the jungle, mountains, or grasslands. It is thought that the awakened memories in ancient times help us fall into sleep faster. Meditation helps people relax and rapidly fall asleep by distracting attention and reducing anxiety (Martires and Zeidler, 2015).

*Light therapy* is gaining increasing attention from doctors and hospitals. The principle of light therapy is to adjust the phase and amplitude of biological clock oscillation through specific light stimulation, so as to establish and consolidate a regular sleep–wake cycle and improve the quality of sleep (van Maanen et al., 2016). Light therapy products have been developed in the form of large light boards and small desktop lightboxes. Recently, head-mounted light therapy glasses have been invented to improve portability and ease of use. The innovation has dramatically increased the patients’ compliance with light therapy.

## Discussion and Conclusion

Having a better knowledge on sleep physiology and insomnia is important for both health and medical reasons. Although we lack further understanding of the nerve system controlling the sleep–wake cycle, it doesn’t stop us from seeking better monitoring and treatment on insomnia.

The pre-programmed passive wearables provide us a new angle to understand sleep, together with the classical evaluation form and PSG, shaping a comprehensive monitoring ecosphere and potential synergistic effect for clinical, post-hospital, and daily needs.

On the other hand, we need a better solution to improve sleep disorder, and there has been progress showing that, besides sleep medications, we do have more effective choices owing to the development of non-drug insomnia therapeutic.

The availability of digitized data in clinical and daily scenarios, combined with the arrival of powerful artificial intelligence (AI) algorithms, could bring deeper implications for health and medicine industry. New medicine or non-drug equipment like light therapy will speed up the upgrade.

The health innovation system should promote such progress by working closely with hospitals, pharmaceutical companies, and academic institutes. Proper guidance should be given to educate the public on the limitations along with the promotion of health technologies.

## Author Contributions

WC and JC: first draft. WC: literature. YG: modified. JC: proofreading.

## Conflict of Interest

WC and JC were employed by the company Shenzhen Qianhai Icecold IT Co., Ltd. The remaining author declares that the research was conducted in the absence of any commercial or financial relationships that could be construed as a potential conflict of interest.

## References

[B1] Ancoli-Israel S. Roth T. (1999). Characteristics of insomnia in the United States: results of the 1991 national sleep foundation survey. I. *Sleep* 22(Suppl. 2), S347–S353. 10394606

[B2] Boulos M. I. Jairam T. Kendzerska T. Im J. Mekhael A. Murray B. J. (2019). Normal polysomnography parameters in healthy adults: a systematic review and meta-analysis. *Lancet Respir. Med.* 7 533–543. 10.1016/S2213-2600(19)30057-8 31006560

[B3] Brown R. E. Basheer R. McKenna J. T. Strecker R. E. McCarley R. W. (2012). Control of sleep and wakefulness. *Physiol. Rev.* 92 1087–1187. 10.1152/physrev.00032.2011 22811426 PMC3621793

[B4] Buysse D. J. (2013). Insomnia. *JAMA* 309 706–716. 10.1001/jama.2013.193 23423416 PMC3632369

[B5] Buysse D. J. Reynolds C. F. III Monk T. H. Berman S. R. Kupfer D. J. (1989). The Pittsburgh Sleep Quality Index: a new instrument for psychiatric practice and research. *Psychiatry Res.* 28 193–213. 10.1016/0165-1781(89)90047-4 2748771

[B6] Cao X. L. Wang S. B. Zhong B. L. Zhang L. Ungvari G. S. Ng C. H. (2017). The prevalence of insomnia in the general population in China: a meta-analysis. *PLoS One* 12:e0170772. 10.1371/journal.pone.0170772 28234940 PMC5325204

[B7] Claudino J. G. de Sa Souza H. Simim M. Fowler P. de Alcantara Borba D. Melo M. (2019). Which parameters to use for sleep quality monitoring in team sport athletes? A systematic review and meta-analysis. *BMJ Open Sport Exerc. Med.* 5:e000475. 10.1136/bmjsem-2018-000475 30729029 PMC6340585

[B8] Espana R. A. Scammell T. E. (2004). Sleep neurobiology for the clinician. *Sleep* 27 811–820. 15283019

[B9] Grandner M. A. Kripke D. F. Yoon I. Y. Youngstedt S. D. (2006). Criterion validity of the pittsburgh sleep quality index: investigation in a non-clinical sample. *Sleep Biol. Rhythms* 4 129–139. 22822303 10.1111/j.1479-8425.2006.00207.x PMC3399671

[B10] Gregorio M. G. Jacomelli M. Inoue D. Genta P. R. de Figueiredo A. C. Lorenzi-Filho G. (2011). Comparison of full versus short induced-sleep polysomnography for the diagnosis of sleep apnea. *Laryngoscope* 121 1098–1103. 10.1002/lary.21658 21520130

[B11] Harvey C. J. Gehrman P. Espie C. A. (2014). Who is predisposed to insomnia: a review of familial aggregation, stress-reactivity, personality and coping style. *Sleep Med. Rev.* 18 237–247. 10.1016/j.smrv.2013.11.004 24480386

[B12] Hood H. K. Rogojanski J. Moss T. G. (2014). Cognitive-behavioral therapy for chronic insomnia. *Curr. Treat. Options Neurol.* 16:321. 10.1007/s11940-014-0321-6 25335933

[B13] Horne J. A. Ostberg O. (1976). A self-assessment questionnaire to determine morningness-eveningness in human circadian rhythms. *Int. J. Chronobiol.* 4 97–110. 1027738

[B14] Jafari B. Mohsenin V. (2010). Polysomnography. *Clin. Chest. Med.* 31 287–297. 10.1016/j.ccm.2010.02.005 20488287

[B15] Jiang C. G. Zhang T. Yue F. G. Yi M. L. Gao D. (2013). Efficacy of repetitive transcranial magnetic stimulation in the treatment of patients with chronic primary insomnia. *Cell Biochem. Biophys.* 67 169–173. 10.1007/s12013-013-9529-4 23797608

[B16] Johnson E. O. Roth T. Schultz L. Breslau N. (2006). Epidemiology of DSM-IV insomnia in adolescence: lifetime prevalence, chronicity, and an emergent gender difference. *Pediatrics* 117:e00247-56. 16452333 10.1542/peds.2004-2629

[B17] Kapur V. K. Auckley D. H. Chowdhuri S. Kuhlmann D. C. Mehra R. Ramar K. (2017). Clinical practice guideline for diagnostic testing for adult obstructive sleep apnea: an american academy of sleep medicine clinical practice guideline. *J. Clin. Sleep Med.* 13 479–504. 10.5664/jcsm.6506 28162150 PMC5337595

[B18] Kelly J. M. Strecker R. E. Bianchi M. T. (2012). Recent developments in home sleep-monitoring devices. *ISRN Neurol.* 2012:768794. 10.5402/2012/768794 23097718 PMC3477711

[B19] Kosmadopoulos A. Sargent C. Darwent D. Zhou X. Roach G. D. (2014). Alternatives to polysomnography (PSG): a validation of wrist actigraphy and a partial-PSG system. *Behav. Res. Methods* 46 1032–1041. 10.3758/s13428-013-0438-7 24442593

[B20] Kryger M. H. (2006). The burden of chronic insomnia on society. *Manag. Care* 15(9 Suppl. 6), 1–5. 17175621

[B21] Lee X. K. Chee N. Ong J. L. Teo T. B. van Rijn E. Lo J. C. (2019). Validation of a consumer sleep wearable device with actigraphy and polysomnography in adolescents across sleep opportunity manipulations. *J. Clin. Sleep Med.* 15 1337–1346. 10.5664/jcsm.7932 31538605 PMC6760396

[B22] Martires J. Zeidler M. (2015). The value of mindfulness meditation in the treatment of insomnia. *Curr. Opin. Pulm. Med.* 21 547–552. 10.1097/MCP.0000000000000207 26390335

[B23] Messineo L. Taranto-Montemurro L. Sands S. A. Oliveira Marques M. D. Azabarzin A. Wellman D. A. (2017). Broadband sound administration improves sleep onset latency in healthy subjects in a model of transient insomnia. *Front. Neurol.* 8:718. 10.3389/fneur.2017.00718 29312136 PMC5742584

[B24] Miller C. B. Espie C. A. Epstein D. R. Friedman L. Morin C. M. Pigeon W. R. (2014). The evidence base of sleep restriction therapy for treating insomnia disorder. *Sleep Med. Rev.* 18 415–424. 10.1016/j.smrv.2014.01.006 24629826

[B25] Mollayeva T. Thurairajah P. Burton K. Mollayeva S. Shapiro C. M. Colantonio A. (2016). The Pittsburgh sleep quality index as a screening tool for sleep dysfunction in clinical and non-clinical samples: a systematic review and meta-analysis. *Sleep Med. Rev.* 25 52–73. 10.1016/j.smrv.2015.01.009 26163057

[B26] Morin C. M. (2004). Cognitive-behavioral approaches to the treatment of insomnia. *J. Clin. Psychiatry* 65(Suppl. 16), 33–40. 15575803

[B27] Newell J. Mairesse O. Verbanck P. Neu D. (2012). Is a one-night stay in the lab really enough to conclude? First-night effect and night-to-night variability in polysomnographic recordings among different clinical population samples. *Psychiatry Res.* 200 795–801. 10.1016/j.psychres.2012.07.045 22901399

[B28] Peake J. M. Kerr G. Sullivan J. P. (2018). A critical review of consumer wearables, mobile applications, and equipment for providing biofeedback, monitoring stress, and sleep in physically active populations. *Front. Physiol.* 9:743. 10.3389/fphys.2018.00743 30002629 PMC6031746

[B29] Schutte-Rodin S. Broch L. Buysse D. Dorsey C. Sateia M. (2008). Clinical guideline for the evaluation and management of chronic insomnia in adults. *J. Clin. Sleep Med.* 4 487–504. 10.5664/jcsm.27286 18853708 PMC2576317

[B30] Schwab J. J. Bialow M. R. Clemmons R. S. Holzer C. E. (1967). Hamilton rating scale for depression with medical in-patients. *Br. J. Psychiatry* 113 83–88. 6029372 10.1192/bjp.113.494.83

[B31] Thomas R. J. Mietus J. E. Peng C. K. Goldberger A. L. (2005). An electrocardiogram-based technique to assess cardiopulmonary coupling during sleep. *Sleep* 28 1151–1161. 10.1093/sleep/28.9.1151 16268385

[B32] van Maanen A. Meijer A. M. van der Heijden K. B. Oort F. J. (2016). The effects of light therapy on sleep problems: a systematic review and meta-analysis. *Sleep Med. Rev.* 29 52–62. 10.1016/j.smrv.2015.08.009 26606319

